# Structural insights into the recognition of telomeric variant repeat TTGGGG by broad-complex, tramtrack and bric-à-brac - zinc finger protein ZBTB10

**DOI:** 10.1016/j.jbc.2023.102918

**Published:** 2023-01-16

**Authors:** Suman Wang, Ziyan Xu, Meili Li, Mengqi Lv, Siyuan Shen, Yunyu Shi, Fudong Li

**Affiliations:** MOE Key Laboratory for Cellular Dynamics, The School of Life Sciences, Division of Life Sciences and Medicine, University of Science and Technology of China, Hefei, Anhui, China

**Keywords:** ZBTB10, C2H2, telomere, TZAP, ZBTB48, ITC, isothermal titration calorimetry, ZBTB10, zinc finger and BTB domain–containing protein 10, ZF, zinc finger

## Abstract

Multiple proteins bind to telomeric DNA and are important for the role of telomeres in genome stability. A recent study established a broad-complex, tramtrack and bric-à-brac - zinc finger (BTB-ZF) protein, ZBTB10 (zinc finger and BTB domain–containing protein 10), as a telomeric variant repeat–binding protein at telomeres that use an alternative method for lengthening telomeres). ZBTB10 specifically interacts with the double-stranded telomeric variant repeat sequence TTGGGG by employing its tandem C2H2 zinc fingers (ZF1–2). Here, we solved the crystal structure of human ZBTB10 ZF1–2 in complex with a double-stranded DNA duplex containing the sequence TTGGGG to assess the molecular details of this interaction. Combined with calorimetric analysis, we identified the vital residues in TTGGGG recognition and determined the specific recognition mechanisms that are different from those of TZAP (telomere zinc finger–associated protein), a recently defined telomeric DNA–binding protein. Following these studies, we further identified a single amino-acid mutant (Arg767Gln) of ZBTB10 ZF1–2 that shows a preference for the telomeric DNA repeat TTAGGG sequence. We solved the cocrystal structure, providing a structural basis for telomeric DNA recognition by C2H2 ZF proteins.

Telomeres, nucleoprotein structures at the end of linear chromosomes, consist of tandem telomeric DNA repeats that are bound by a variety of proteins. For vertebrates, the nucleotide sequence of telomeric DNA repeats is TTAGGG (G-strand), with the complementary strand being CCCTAA (C-strand). The G-strand is longer than the C-strand, forming a single-stranded overhang that invades the double-stranded region, an architecture named the T-loop (1, 2). Moreover, telomeric DNA is bound by diverse proteins, among which a six-subunit protein complex called shelterin is the most dominant (3). Shelterin is composed of TRF1, TRF2, POT1, TPP1, TIN2, and Rap1. TRF1/2 and POT1 directly interact with double-stranded and single-stranded telomeric DNA, respectively (4). Shelterin, together with other proteins that it recruits, helps to shape the telomeric DNA structure and contributes to telomere function in protecting linear chromosome ends and genome stability (5, 6, 7).

Although more than 200 proteins act on telomeres, proteins that directly bind double-stranded telomeric DNA have been restricted to the shelterin subunits TRF1/2 and a protein called HOT1 during the past 2 decades. Recently, two groups identified a BTB-ZF protein, ZBTB48 (renamed TZAP [telomere zinc finger–associated protein] for telomeric zinc finger [ZF]–associated protein). TZAP binds double-stranded telomeric DNA directly and stimulates telomere trimming, a process that is a regulated form of telomere rapid deletion (8, 9). TZAP is a 688-amino acid protein that comprises an N-terminal BTB domain and 11 adjacent C2H2-type ZFs (ZF1–11) at its C terminus. We reported the structure of TZAP in complex with the telomeric DNA sequence and found that TZAP employed ZF11 and a conserved C-terminal loop to specifically recognize telomeric DNA (10). These works clearly demonstrated that the C2H2 finger can bind telomeric DNA, while having a completely different protein domain type from the homeodomain that TRF1/2 and HOT1 employ to recognize.

More than 700 human genes encode C2H2 ZF proteins, and these proteins play essential roles in gene expression regulation (11). However, the specific DNA targets of most C2H2 proteins remain elusive (12). The identification of TZAP as a telomere-binding protein suggests that more C2H2 finger proteins play roles in telomere functions. Very recently, another BTB-ZF protein, ZBTB10 (zinc finger and BTB domain–containing protein 10), has been reported to directly bind a telomeric variant DNA sequence, TTGGGG (13, 14). Telomeric variant repeats can be found in subtelomere regions, which are segments of DNA between telomeric caps and chromatin (15). They can also be found in the telomeric region of cells that use an alternative lengthening of telomeres mechanism (16, 17), which is based on homology-directed repair (18).

Previous studies have established a conventional C2H2 ZF DNA recognition code (one finger-three base rule) (19, 20) in which three amino acids at three canonical “recognition” positions in each finger mediate specific DNA base contacts. Furthermore, variant numbers of such ZFs could be linked tandemly to recognize DNA of varying length (21). According to the one finger-three base rule, the C2H2 ZF requires at least two fingers to recognize telomeric hexamer DNA. However, TZAP employs only ZF11 to recognize the GGG sequence, while a conserved C-terminal arm is inserted into the minor groove to recognize the TA sequence (10). Unlike TZAP, ZBTB10 requires two ZF domains to fully recognize the TTGGGG sequence, which represents a different mode of telomeric or telomeric variant DNA binding.

In this study, we present the structure of ZBTB10 ZF1–2 in complex with a double-stranded oligo containing the TTGGGG sequence. Our structure reveals that ZF1–2 fits into the major groove, making plentiful contacts with mainly the G strand but also with the C strand. ZF2 specifically recognizes the GGG sequence with residues occurring at positions −1, −4, and −7, when the first zinc-coordinating histidine was numbered 0 as described by the Xiaodong Cheng group (22). ZF1 recognizes the TTG sequence in a similar way. Furthermore, when we mutated Arg767 to Gln, the mutant showed a binding preference for TTAGGG over TTGGGG. We further determined the structure of the Arg767Gln mutant in complex with the TTAGGG sequence. The structure shows that Gln767 specifically recognizes adenine by forming a bidentate H-bond interaction. These data imply that C2H2 fingers can employ distinct modes to recognize telomeric DNA, as revealed by the complex structures of TZAP-DNA and ZBTB10-DNA. These results also led us to propose that there could be more C2H2 finger proteins that could act on telomeres, which requires further investigation.

## Results

### Overall structure of human ZBTB10 ZF1–2 in complex with the TTGGGG sequence

**Figure 1:**
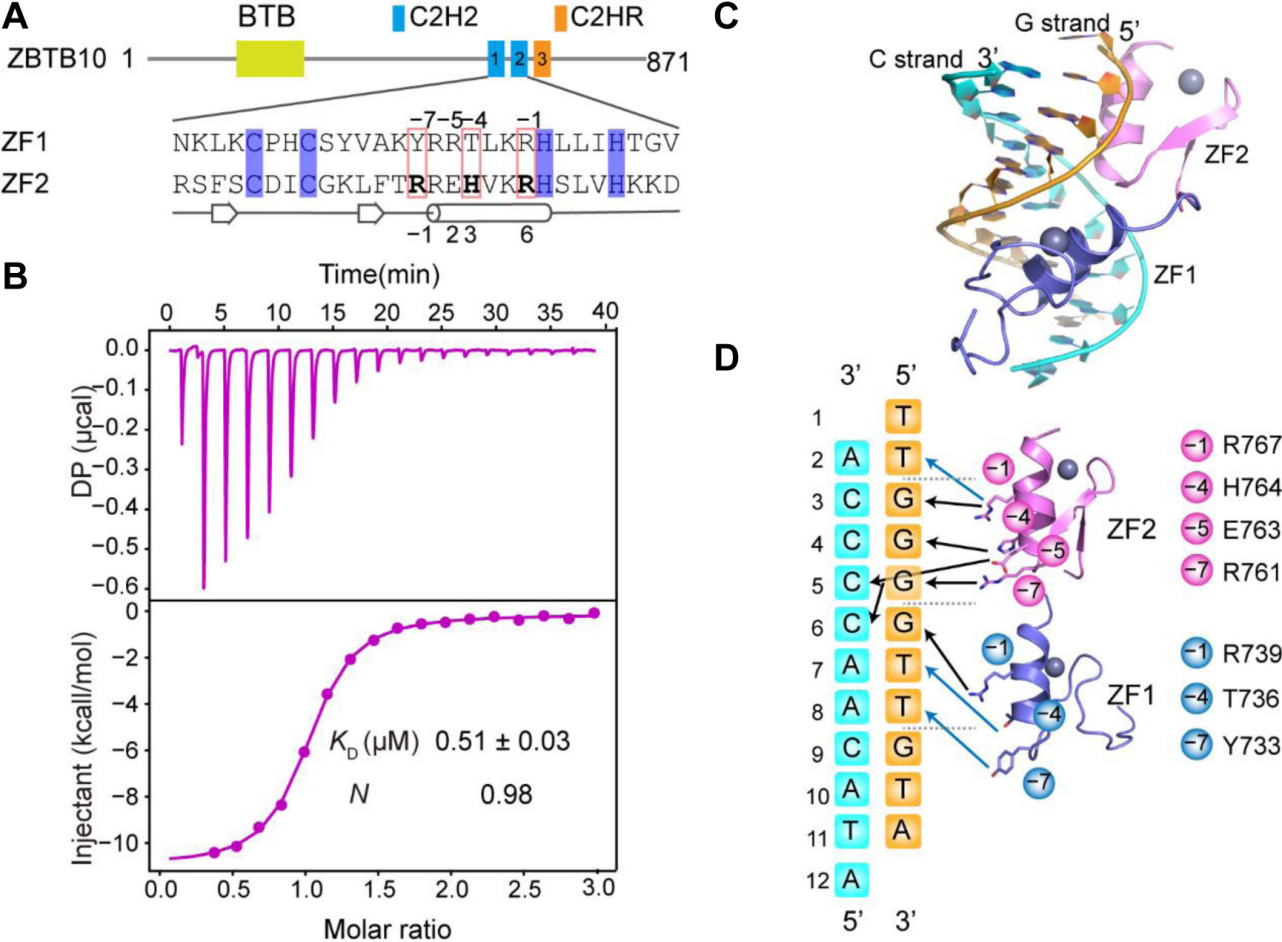
**Overall structure of ZBTB10 ZF1–2 in complex with the telomeric variant repeat TTGGGG.***A*, schematic representation of the domain architecture of ZBTB10 (*top panel*). The sequence of ZF1–2 together with the secondary structure is shown. Two cysteine and two histidine residues in each finger are responsible for Zn^2+^ binding (*slate*). Three residues in ZF1 and ZF2 interact specifically with the DNA bases (*pink boxes*). *B*, ITC data show that ZBTB10 ZF1–2 binds to the TTGGGG duplex sequence with a high affinity. *C*, the overall structure of ZF1–2 of ZBTB10 in complex with the TTGGGG sequence is shown in *cartoon* representation. ZF1 and ZF2 are colored *slate* and *violet*, respectively. The G-strand and C-strand are colored *orange* and *cyan*, respectively. *D*, schematic diagram of detailed base-specific interactions between ZBTB10 ZF1–2 and the 11 bp TTGGGG sequence. Color codes of ZF1, ZF2, and DNA sequence are defined as in *C*. ITC, isothermal titration calorimetry; ZBTB10, zinc finger and BTB domain–containing protein 10; ZF, zinc finger.

Previous studies showed that both ZF1 and ZF2 of ZBTB10 were essential to recognize the telomeric variant TTGGGG motif, and a C-terminal adjacent C2HR further promoted the binding affinity (13). C2HR motifs are C2H2 motifs in which the last His has been replaced by Arg. To investigate the molecular mechanism by which ZBTB10 recognizes the telomeric variant motif TTGGGG, we tried to crystallize two protein constructs, ZF1–2 and ZF1–2-C2HR (Fig. 1*A*). The two proteins were cocrystallized with a variety of double-stranded DNA duplexes with different sequences, lengths, and overhangs. Finally, ZBTB10 ZF1–2 (amino acids 713–779) in complex with an 11-base-pair (bp) oligonucleotide containing the TTGGGG sequence was successfully crystallized. The G-strand sequence of the oligo is “TTGGGGTTGTA,” and the C-strand sequence is “ATACAACCCCA,” plus a 5′-overhang thymine on the G-strand and a 5′-overhang adenine on the C-strand to facilitate crystal packing (Table S1). We further measured the binding affinity between ZF1–2 and the DNA sequence without the overhangs, using isothermal titration calorimetry (ITC). The results showed that ZBTB10 ZF1–2 binds the DNA duplex with a *K*_*D*_ value of ∼0.51 μM and a 1:1 stoichiometry (Fig. 1*B* and Table 1).

**Table 1:** ITC results

Protein	DNA	△H (kcal/mol)	−T△S kcal/mol	N	*K*_*D*_ (mM)
WT	TTGGGGTTG	−10.8 ± 0.11	2.38	0.98	0.51 ± 0.03
R761A	TTGGGGTTG				ND
H764A	TTGGGGTTG				>10
R767A	TTGGGGTTG				>10
E763A	TTGGGGTTG	−7.9 ± 0.23	0.31	0.92	2.05 ± 0.23
Y733A	TTGGGGTTG	−14.6 ± 0.33	6.49	0.95	0.94 ± 0.12
Y733G	TTGGGGTTG	−11.3 ± 0.31	3.68	0.97	2.02 ± 0.22
T736A	TTGGGGTTG	−9.1 ± 0.18	1.21	1.04	1.44 ± 0.14
R739A	TTGGGGTTG	−8.9 ± 1.51	2.19	1.00	8.87 ± 0.32
WT	TT**A**GGGTTG				>10
WT	TTG**A**GGTTG				>10
WT	TTGG**A**GTTG				>10
WT	TTGGG**A**TTG				>10
WT	TTGGGG**C**TG	−6.0 ± 0.18	−1.59	0.94	2.13 ± 0.25
R767Q	TT**A**GGGTTG	−12.8 ± 0.18	5.70	0.91	4.75 ± 0.19
R767Q	TTGGGGTTG				>10
R767N	TT**A**GGGTTG				>10
R767N	TTGGGGTTG				>10

Abbreviation: ND, not detectable binding.

>10: represents that the fitting *K*_*D*_ value is above 10 μM.

We determined the structure of the protein‒DNA complex in the *P*2_1_2_1_2_1_ group to 1.8 Å resolution (Table S2), with the *R*_work_ and *R*_free_ being refined to 20.00% and 22.50%, respectively. In each crystallographic asymmetric unit, there are two ZF1–2 molecules (chains A and B), each binding a DNA duplex, consistent with the 1:1 stoichiometry provided by the ITC results. These two ZF1–2 molecules are highly similar to each other (with an RMSD of 0.506 Å over 49 aligned Cα atoms), except for the very N-terminal five residues, which seem to be flexible. Both ZF1 and ZF2 adopt a canonical C2H2 ZF fold, which consists of two β-strands and one C-terminal α-helix, coordinating one zinc ion (23). ZF1–2 fits into the major groove of the DNA duplex, which shows a B-DNA conformation. We numbered the 11-bp oligo 1 to 11 from 5′–3′ of the G-strand, and the ZF1–2 protein sequence runs in the opposite direction of the G-strand (Fig. 1*C*). The overall structure showed that each ZF recognizes approximately 3 bp of the DNA duplex, with ZF2 mainly interacting with G_3_G_4_G_5_ and ZF1 with G_6_T_7_T_8_ sequences. Thus, ZBTB10 ZF1–2 makes base-specific contacts with all six base pairs of the TTGGGG sequence, contributing to high specificity (Fig. 1*D*). Moreover, similar to other C2H2–DNA complexes, ZBTB10 ZF1–2 makes substantial phosphate contacts with the DNA backbones, enhancing the binding affinity.

### Base-specific recognition provided by ZF2

**Figure 2:**
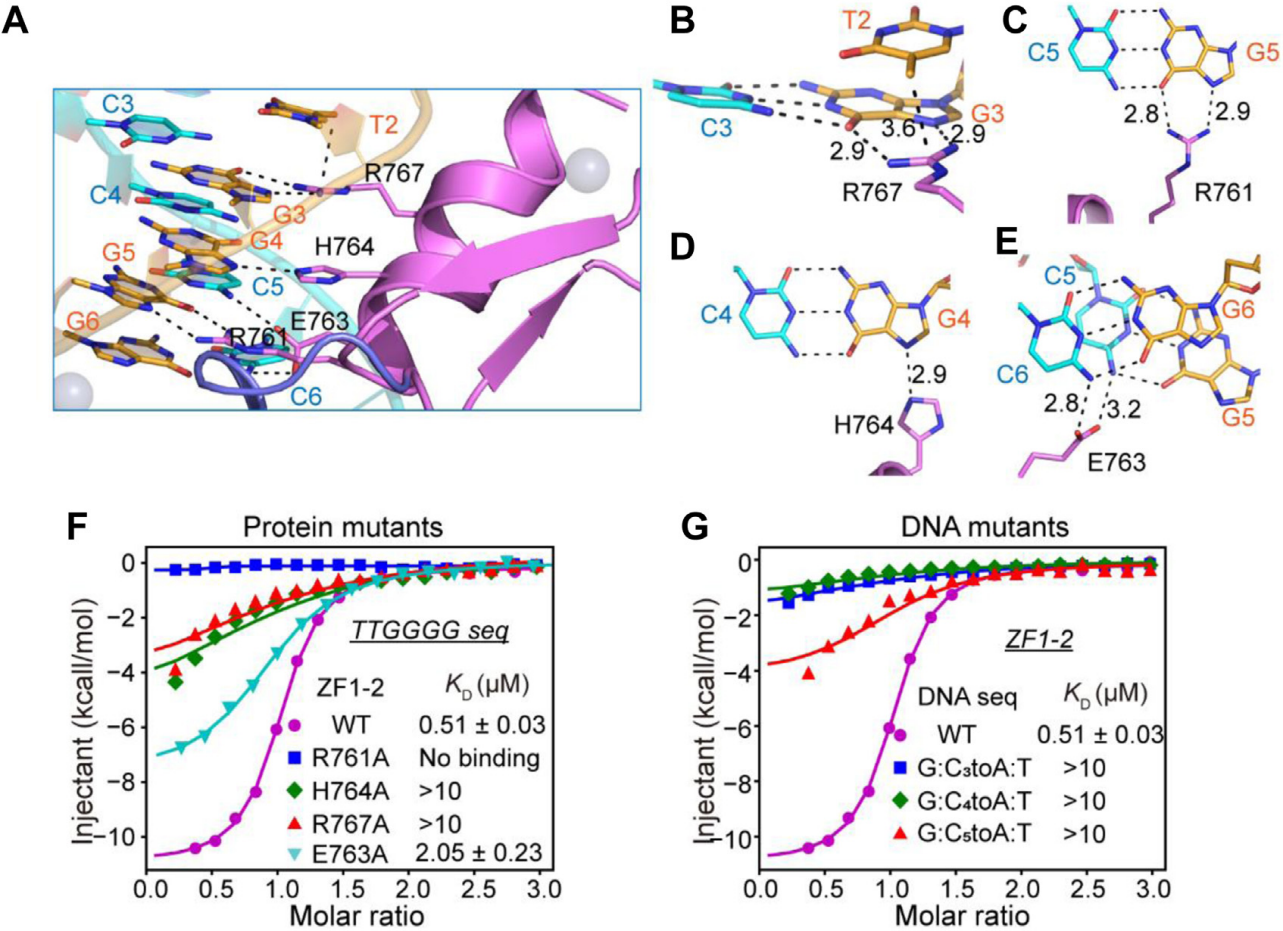
**Base-specific recognition provided by ZBTB10 ZF2.***A*–*E*, details of ZBTB10 ZF2-G_3_G_4_G_5_ triplet base-specific interactions. The hydrogen bonds are depicted as *black dashed lines*. *F*, the effects of ZF2 mutations on TTGGGG binding, measured *via* ITC. *G*, mutating each base pair at positions 3, 4, and 5 to the A:T pair reduced the binding by ZF2. ITC, isothermal titration calorimetry; ZBTB10, zinc finger and BTB domain–containing protein 10; ZF, zinc finger.

ZF2 makes base-specific contacts with the G_3_G_4_G_5_ nucleotide triplet. The positions −1, −4, and −7 residues (Arg767, His764, and Arg761, respectively) make direct hydrogen-bonding interactions with G_3_, G_4_, and G_5_ on the G-strand (Fig. 2*A*). Arg767 and Arg761 recognize guanine in a similar manner, with their terminal Nη1 and Nη2 groups donating hydrogen bonds to guanine O6 and N7 atoms, respectively (Fig. 2, *B* and *C*). Such an Arg-Gua recognition mode is commonly found in other C2H2 fingers or DNA-binding modules (24, 25). His764 specifically recognizes G_4_, with its Nε2 group donating one hydrogen bond to the N7 atom of G_4_ (Fig. 2*D*). Histidine emerged as the most common ZF residue at position −4 to recognize nucleotides in a high-throughput bacterial one-hybrid study (12). In addition to these base-specific recognitions on the G-strand, we also found that the position −5 residue, Glu763, contacts C_5_ and C_6_ on the C-strand, with its side-chain carboxyl group hydrogen bonding to N4 atoms of both C_5_ and C_6_ (Fig. 2*E*). These hydrogen-bonding interactions confer specific recognition of the G_3_G_4_G_5_ triplet. In addition to the recognition of G_3_, we observed that Arg767 interacts with T2, with its guanidino group making van der Waals contacts with the C5 methyl group of T_2_ (Fig. 2*B*). Thus, Arg767 together with G3 and T2 form a methyl–Arg–Gua triad recognition, which can also be found in other C2H2 fingers (such as ZFP57), methyl-binding domains, and p53 protein, facilitating recognition of 5mCpG or TpG (26, 27, 28, 29).

The importance of these interactions for the binding of ZF2 to the G_3_G_4_G_5_ triplet was confirmed by mutational analysis. ZF1–2 with any of the mutations, Arg767Ala, Arg761Ala, His764Ala, or Glu763Ala, caused severely reduced affinities for the TTGGGG probe in ITC assays (Fig. 2*F* and Table 1). Accordingly, when we mutated each G:C pair to an A:T base pair, these changes also resulted in significantly reduced binding affinities to wildtype ZBTB10 ZF1–2 (Fig. 2*G* and Table 1).

### Base-specific recognition provided by ZF1

**Figure 3:**
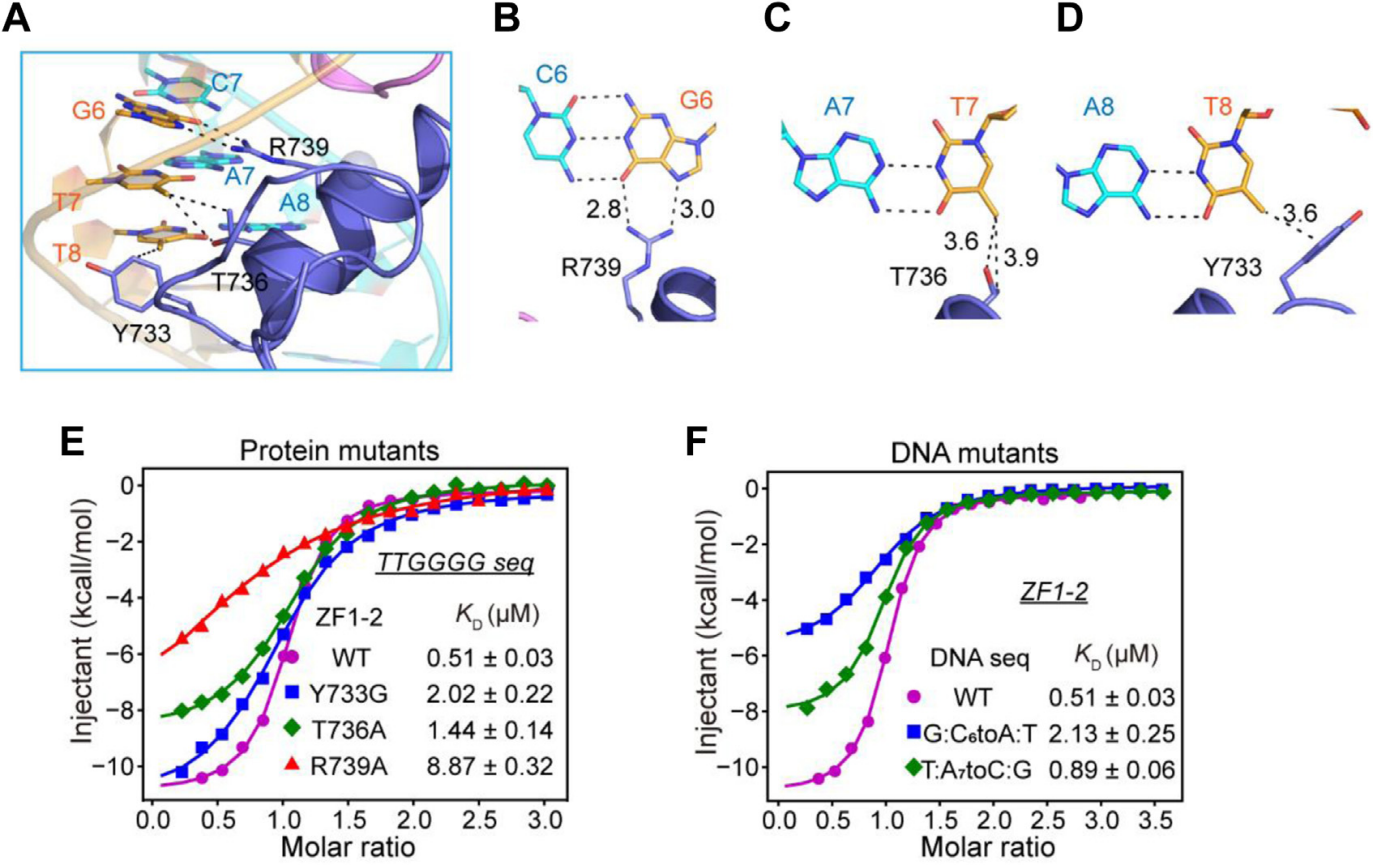
**Base-specific recognition provided by ZBTB10 ZF1.***A*–*D*, details of ZBTB10 ZF1-G_6_T_7_T_8_ triplet base-specific interactions. The hydrogen bonds and van der Waals interactions are depicted as *black dashed lines*. *E*, the effects of ZF1 mutations on TTGGGG binding. *F*, mutating each base pair at positions 6 and 7 to the A:T pair reduced the binding by ZF1. ZBTB10, zinc finger and BTB domain–containing protein 10; ZF, zinc finger.

The G_6_T_7_T_8_ triplet is recognized mainly by ZF1. Similar to the recognition pattern of G_3_ and G_5_, G_6_ accepts two hydrogen bonds from the side chain of Arg739, the −1 residue of ZF1 (Fig. 3, *A* and *B*). Accordingly, the binding affinity of the Arg739Ala mutant for the TTGGGG probe was significantly reduced (Fig. 3*E*). Consistently, when the G:C_6_ pair was substituted with an A:T pair, the binding affinity was substantially reduced, further confirming the importance of the hydrogen-bonding contacts (Fig. 3*F*). Unlike the G_3_G_4_G_5_G_6_ tetrad, which was specifically recognized through hydrogen-bonding contacts, we observed that T_7_ and T_8_ are recognized mainly by van der Waals contacts. The side chain of the −4 residue of ZF1, Thr736, forms van der Waals contacts with the C5 methyl group of T_7_ (Fig. 3*C*). Consistently, the mutation of Thr736 to alanine (Thr736Ala) decreased the binding affinity by approximately 2.8-fold (Fig. 3*E* and Table 1). The substitution of the T:A pair with C:G pair, which would abolish the van der Waals contacts, also decreased the binding affinity by approximately 1.8-fold (Fig. 3*F* and Table 1). The recognition of T_8_ was also achieved by van der Waals contacts, and we observed that the C6 methyl group of T_8_ contacts the side chains of both Thr736 and Tyr733 (−7 residue of ZF1) in chain A (Fig. 3*D*). Although the electronic density of the side chain of Tyr733 could not be clearly seen in chain B, ZF1–2 Tyr733Ala and Tyr733Gly bound to the DNA probe with approximately 1.8- and 4-fold reduced affinity compared with that of wildtype ZF1–2, respectively (Fig. 3*E* and Table 1). These data indicate that Tyr733 and Thr736 play important roles in the recognition of T_8_ and T_7_ by forming van der Waals contacts.

### Arg767Gln mutant preferentially binds the telomeric TTAGGG sequence

**Figure 4:**
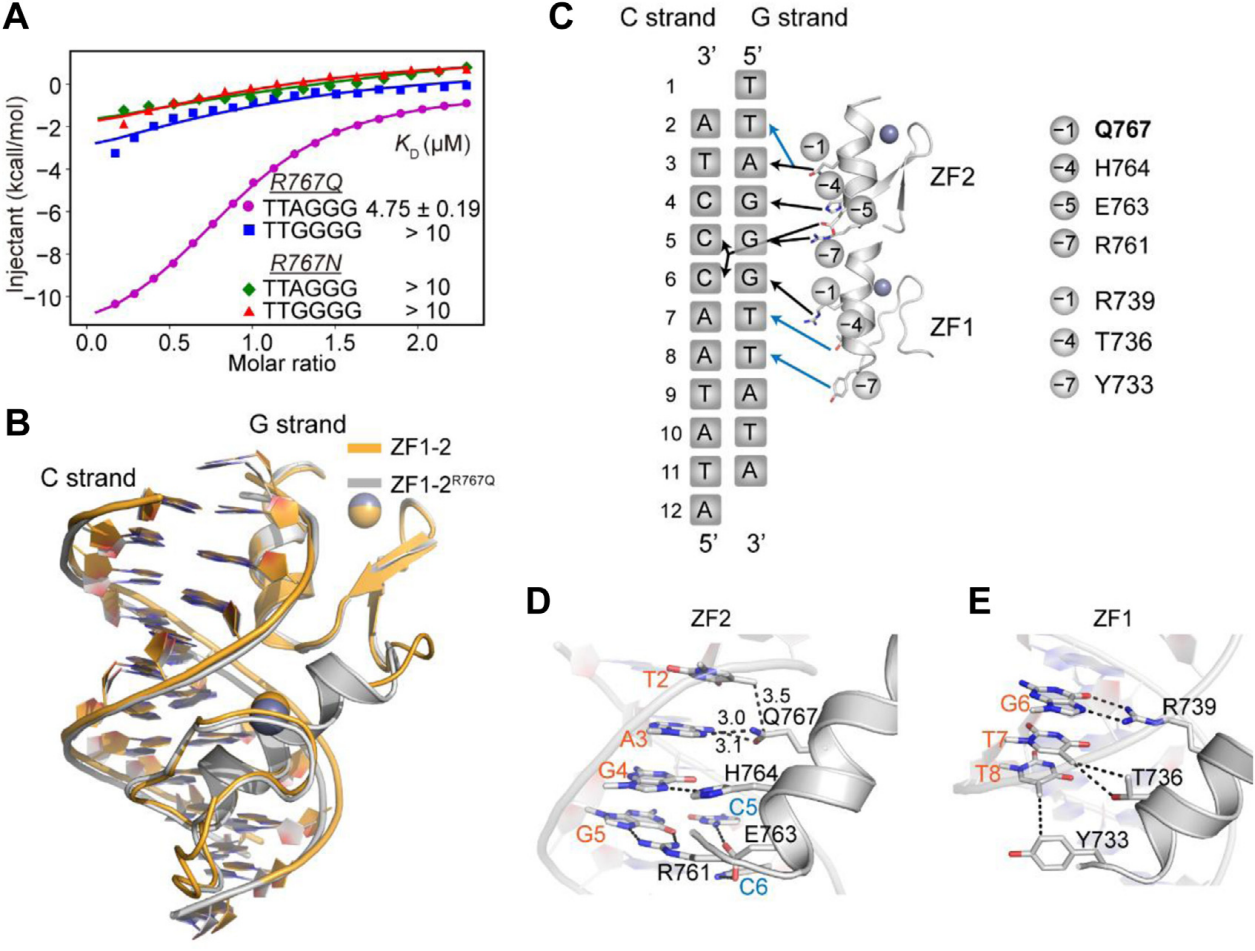
**The ZBTB10 Arg767Gln mutant preferentially binds the telomeric TTAGGG sequence.***A*, comparison of the binding affinities of ZBTB10 ZF1–2^R767Q^/ZBTB10 ZF1–2^R767N^ with the TTAGGG and TTGGGG DNA sequences, respectively. *B*, overlaid structures of ZBTB10 ZF1–2 and ZBTB10 ZF1–2^R767Q^ bound to TTGGGG DNA. The ZBTB10 ZF1–2:TTGGGG complex and ZBTB10 ZF1–2^R767Q^:TTAGGG complex are colored *orange* and *gray*, respectively. *C*, schematic diagram of detailed base-specific interactions between ZBTB10 ZF1–2^R767Q^ and the TTAGGG sequence. *D* and *E*, details of ZBTB10 ZF1–2^R767Q^ recognizing the TTGGGG DNA sequence. The hydrogen bonds and van der Waals contacts are depicted as *black dashed lines*. ZBTB10, zinc finger and BTB domain–containing protein 10; ZF, zinc finger.

ZBTB10 ZF1–2 recognizes the TTGGGG sequence in a different manner from which TZAP recognizes the TTAGGG telomeric sequence, which employs a C-terminal loop inserted into the minor groove (10). Next, we attempted to engineer ZF1–2 of ZBTB10 to recognize the TTAGGG sequence. Previous studies have revealed that the juxtaposition of Asn or Gln with adenine is a generic mechanism for recognition of the adenine base (12, 20, 24, 30). Based on the structural mechanism by which ZBTB10 recognizes the TTGGGG sequence, we rationally mutated Arg767 to either asparagine or glutamine and performed ITC assays to check their DNA-binding affinities. We found that the Arg767Asn mutant resulted in severely reduced affinities for both the TTAGGG and TTGGGG sequences. However, the Arg767Gln mutant (referred to as ZBTB10 ZF1–2^R767Q^) bound to the TTAGGG probe with an obviously higher binding affinity compared with the TTGGGG probe, indicating its preference for telomeric DNA binding (Fig. 4*A* and Table 1).

Next, we solved the structure of ZBTB10 ZF1–2^R767Q^ in complex with the TTAGGG sequence. The G-strand sequence of the oligo is “TTAGGGTTATA,” and the C-strand sequence is “ATATAACCCTA” (Table S1). We determined the structure in the *P*1 space group to 1.9 Å resolution (Table S2), with one ZBTB10 ZF1–2^R767Q^ protein binding one DNA duplex in each crystallographic asymmetric unit. ZBTB10 ZF1–2^R767Q^ shows high similarity to wildtype ZF1–2 with a RMSD of 0.586 Å over 49 Cα atoms (Fig. 4*B*). We observed that the recognition pattern of G_4_G_5_G_6_T_7_T_8_ was preserved very well (Fig. 4, *C*, *D*, and *E*). Furthermore, Gln767 expectedly donates one hydrogen bond to the adenine N7 atom and accepts one hydrogen bond from the N6 atom of A_3_, thus conferring the preference of TTAGGG by the Arg767Gln mutant (Fig. 4*D*). Taken together, we engineered a tandem C2H2 finger protein to recognize the telomeric TTAGGG sequence based on ZBTB10 ZF1–2.

### The Arg–His–Arg triad is commonly employed to recognize the Gua–Gua–Gua nucleotide triplet in C2H2 finger proteins

**Figure 5:**
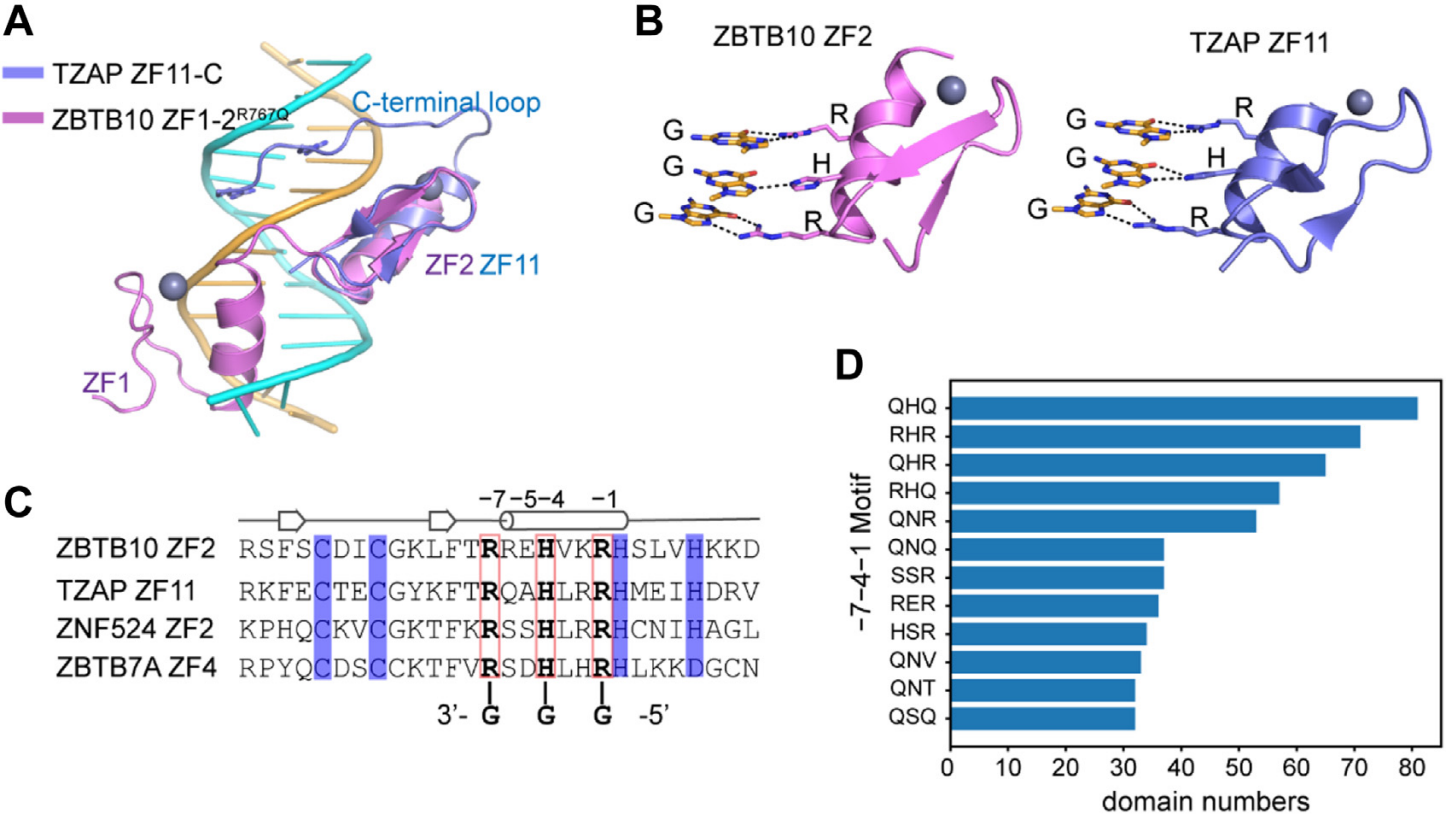
The **Arg–His–Arg triad is commonly employed to recognize the Gua–Gua–Gua nucleotide triplet in C2H2 finger proteins.***A,* ZBTB10 ZF2^R767Q^ (violate) and TZAP ZF11 (slate) recognize telomeric DNA sequences in different manners. *B*, ZBTB10 and TZAP both have a conserved C2H2 finger (Arg(−7)–His(−4)–Arg(−1)) to make base-specific contacts with a Gua–Gua–Gua DNA triplet. *C*, sequence alignment shows that some C2H2 fingers possessing the Arg(−7)–His(−4)–Arg(−1) triad are potential telomeric DNA binding proteins. *D*, the RHR motif emerges as the second-most-frequent −7−4−1 motif, consistent with the possibility that the Arg(−7)–His(−4)–Arg(−1) triad is frequently employed to recognize the Gua–Gua–Gua DNA triplet in the human genome. ZBTB10, zinc finger and BTB domain–containing protein 10; ZF, zinc finger.

The TZAP-telomeric DNA cocrystal structure demonstrated that TZAP recognizes telomeric DNA sequence elements in a bipartite manner, with its ZF11 and a conserved C-terminal loop contacting the major and minor grooves of its target DNA site, respectively. The phenomenon that the C2H2 domain combined with an N-terminal or C-terminal loop is gradually being recognized as a mechanism to expand their binding specificity (31, 32). Nevertheless, ZBTB10 ZF1–2 and the R767Q mutant recognize telomeric DNA sequences in a distinct mode, with both ZFs making major groove contacts with the DNA site, which follows the well-defined one finger-three base rule (Fig. 5*A*).

Despite their overall different recognition modes, ZBTB10 and TZAP share a similar strategy to recognize a Gua–Gua–Gua nucleotide triplet by employing a C2H2 finger containing the Arg(−7)–His(−4)–Arg(−1) amino acid triad (Fig. 5, *B* and *C*). A previous phylointeractomics screen indicated that there are more C2H2 fingers, including ZBTB7A, ZNF276, ZNF524, ZNF827, VEZF1, and KLF12, which directly bind to telomeric DNA with unknown function (14). An inspection of their amino acid sequences revealed that ZF2 of ZNF524 and ZF4 of ZBTB7A both possess the Arg(−7)–His(−4)–Arg(−1) triad, implying that they are potential telomeric DNA–binding proteins (Fig. 5*C*). Recently, we demonstrated that ZNF524 directly interacts with telomeric DNA and supports telomere integrity, with its ZF2 playing a vital role in telomeric DNA recognition (33). Furthermore, our recent X-ray structures of ZBTB7A ZF1–4 in complex with the −200 sequence element of the γ-globin gene promoter reveal that its ZF4 recognizes a 3′ C:G nucleotide triplet (34). However, whether ZBTB7A directly binds to telomeric DNA requires further study.

There are more than 700 C2H2 finger proteins, with over 6000 C2H2 domains in the human genome, per the SMART database (35). We asked whether the Arg(−7)–His(−4)–Arg(−1) triad is a prevalent motif, given its preference for the GGG motif. We counted the type frequency of the −7−4−1 triad motif, which showed that all the C2H2 domains in the human genome have over 2000 types of −7−4−1 motifs, indicating widespread binding preference of the C2H2 fingers. This binding preference enables C2H2 finger proteins to play important roles in a diverse range of genes and pathways, which requires more investigation. Afterward, we calculated the frequency of each −7−4−1 motif. Although the median number of occurrences is 2, 12 types of triplets occur more than 30 times in the human genome C2H2 domains. Among them, the QHQ and RHR motifs emerged as the two top −7−4−1 motifs, occurring 81 and 71 times, respectively (Figs. 5*D* and S1). This suggests that the Arg(−7)–His(−4)–Arg(−1) triplet is frequently employed to recognize the Gua–Gua–Gua DNA triplet in C2H2 proteins. However, whether any proteins among them could specifically localize to telomeres and have any biological functions remains to be determined.

## Discussion

In mammals, TRF1 and TRF2 are two subunits of the shelterin complex, which directly binds the telomeric repeat sequence TTAGGG through their C-terminal homeodomains (36). TRF1 and TRF2 recruit various proteins during the cell cycle, playing essential roles in telomere homeostasis (37). However, decades after the discovery of shelterin, there are rare proteins reported to directly bind telomeric DNA. Thus, it is an open question whether new factors associated with the telomeric repeat region function in telomere biology. Recently, ZBTB48 (TZAP), the BTB-ZF protein family member, emerged as a new player that directly binds double-stranded telomeric DNA and mediates the telomere trimming process *via* an unknown mechanism (8, 9, 38). A TZAP-telomeric DNA cocrystal structure further provided the molecular mechanism underlying the recognition. TZAP specifically recognizes telomeric DNA sequence elements in a bipartite manner, with its 11th C2H2 finger and a conserved C-terminal loop contacting the major and minor grooves of its target DNA site, respectively (10).

In this and our previous related studies, we reasoned that ZBTB10, also a BTB-ZF protein family member, specifically binds to the TTGGGG sequence, one sequence among the telomeric variant repeats (13). However, the manner of ZBTB10 recognition of the TTGGGG sequence differs from that of TZAP. Roughly, ZBTB10 follows the well-defined one finger-three base rule of C2H2 fingers, with each of its two C2H2 fingers recognizing 3- or 4-bp of DNA. Together, ZBTB10 ZF1–2 provides full recognition of all the bases of the TTGGGG sequence.

The discovery that TZAP can bind telomeres, as well as ZBTB10, can recognize telomeric variant repeats, greatly supporting that the C2H2 finger is a new telomeric or telomere variant repeat–binding motif. C2H2 finger proteins are the most abundant transcription factor family in the human genome, possessing over 700 members (39). Whether more C2H2 finger proteins associate with telomeres, in addition to TZAP and ZBTB10, still requires further investigation. In this study, we also designed a ZBTB10 ZF1–2 mutant that shows a preference for the telomeric TTAGGG sequence. Furthermore, the binding specificity is also proven by a cocrystal structure. These results raise the possibility that C2H2 finger proteins can employ the well-defined one finger-three base rule to specifically recognize the TTAGGG sequence, and there may be more C2H2 finger proteins functioning in telomeres. Moreover, we noticed that ZBTB10 and TZAP both have a conserved C2H2 finger, providing the base-specific contacts for the Gua–Gua–Gua DNA triplet, which could be a distinct feature of telomere-binding C2H2 finger proteins. A search in the human genome for C2H2 finger proteins shows that approximately 5% of them contain such Gua–Gua–Gua recognition C2H2 motifs.

In the Arg(−7)–His(−4)–Arg(−1) ZF proteins list, TZAP, ZBTB10, and ZNF524 have already been identified as telomeric or telomere variant repeat–binding proteins. However, whether the other members could directly bind to telomeric region remains unknown (Fig. S1). In addition to the telomeric region, there are also many regions of the genome that contain GGG motifs, and it is that likely these Arg(−7)–His(−4)–Arg(−1) proteins would recognize such sequences. For example, ZBTB7A, a key molecular regulator of fetal globin expression, binds directly to a G-rich fetal globin promoter element at −200 *via* its ZF4 containing the Arg(−7)–His(−4)–Arg(−1) triad (34). Furthermore, ZBTB7A emerged as a telomeric repeat DNA-binding protein in a previous phylointeractomics screen, but its role in telomere biology requires further study. A closer inspection of the Arg(−7)–His(−4)–Arg(−1) proteins list also led us to find a protein called Zkscan3. It has been shown to bind to the consensus sequence 5′-[GT][AG][AGT]GGGG-3′ and acts as a repressor of autophagy (40). Recently, Zkscan3 was found to counteract cellular senescence and affect telomere length (41). These results imply that Zkscan3 is involved in telomere length homeostasis by directly binding to telomeric regions. Nevertheless, the biological functions of the large majority of the Arg(−7)–His(−4)–Arg(−1) ZF proteins are unknown, requiring further study.

The discoveries of TZAP and ZBTB10 open up more questions about how these new players function in telomere biology. TZAP triggers telomere trimming, a process that results in the rapid deletion of telomeric repeats, and thus may set the upper limit of telomere length. However, the function of ZBTB10 has yet to be well defined, since knockout of ZBTB10 has no obvious effect on telomere homeostasis in either telomerase-positive or alternative lengthening of telomere cells. ZBTB10 and TZAP both belong to the BTB-ZF protein family, which has an N-terminal BTB domain (42). The BTB domain is a highly conserved protein‒protein interaction motif and is present in over 200 human BTB proteins (43). The BTB domain can form dimers, and the dimerized BTB further interacts with other proteins to implement its function (44). This is reminiscent of the TRFH domain of TRF1/2, which act as dimers and recruit variant proteins that are imported for telomere homeostasis. Thus, whether and how the BTB domains of TZAP and ZBTB10 play roles in telomere biology needs further study and may strengthen our mechanistic understanding of the C2H2 finger protein acting on telomeres.

## Experimental procedures

### Protein expression and purification

The ZBTB10 ZF1–2 fragment (residues 713–779) and ZF1–2-C2HR fragment (residues 713–804) were amplified from a human brain complementary DNA library and cloned into a modified pET28a vector with an MBP tag at the N terminus. All mutants were generated using a MutantBEST kit (Takara) and verified *via* DNA sequencing. All the proteins were expressed in *Escherichia coli* BL21 (DE3) cells. The cells were cultured at 37 °C in LB medium with an additional 100 μM ZnSO_4_ until the absorbance at 600 nm reached approximately 1.0. Then, the proteins were induced with 0.3 mM IPTG. After induction at 16 °C for 24 h, the cells were harvested and lysed by sonication in buffer A (20 mM Tris–HCl, pH 7.5, 1 M NaCl). Proteins were purified using nickel–nitrilotriacetic acid agarose beads. The MBP tag was removed by treatment with PreScission protease in buffer B (20 mM Tris–HCl, pH 7.5, 500 mM NaCl), and the purified protein was separated from the tag by size-exclusion chromatography on a HiLoad 16/60 Superdex 75 column (GE Healthcare). Purified proteins were dialyzed with buffer C (20 mM Tris–HCl [pH 7.5] and 150 mM NaCl) and concentrated for subsequent analysis.

### Protein‒DNA complex preparation

The synthetic 11-bp single-stranded DNA fragment was dissolved into buffer C, and the two complementary single-stranded DNAs were mixed in equimolar amounts, incubated in a 95 °C water bath for 5 min, and then gradually cooled to room temperature. The annealed double-stranded DNA was mixed with the purified protein at a 1.2:1 ratio and dialyzed into buffer C to form a protein‒DNA complex. Next, the complex was further purified by size-exclusion chromatography on a HiLoad 16/60 Superdex 75 column in buffer C. Finally, the complex was condensed to 1.2 mM in preparation for crystallization.

### Crystallographic experiments

The crystals were grown at 20 °C *via* the hanging-drop vapor diffusion method. The crystals of ZBTB10 ZF1–2 in complex with the TTGGG sequence were grown by mixing 1 μl of the protein‒DNA complex and 1 μl of reservoir buffer (0.1 M sodium Hepes, pH 8.2, 40% v/v PEG 500 MME). The crystals of ZBTB10 ZF1–2^R767Q^ in the TTAGGG complex sequence were grown by mixing 1 μl of the protein‒DNA complex and 1 μl of reservoir buffer (0.1 M sodium cacodylate, pH 6.5, 27% v/v PEG 2000 MME). All the crystals were harvested in their corresponding reservoir buffers supplemented with 25% (v/v) glycerol and frozen in liquid nitrogen.

X-ray diffraction data sets of the crystals were collected at beamline 19U1 at the Shanghai Synchrotron Radiation Facility with a diffraction wavelength of 0.979 Å. The two data sets, including ZBTB10 ZF1–2 and ZF1–2^R767Q^, were indexed, integrated, and scaled by the *HKL*-2000 (HKL Research, Inc) (45) program suite. Next, the crystallographic phases of the ZBTB10 ZF1–2 data sets were determined with the *AutoSol* program in the *PHENIX* suite (National Institutes of Health [General Medicine] and the Phenix Industrial Consortium), with the zinc atom sites found by the SHELX C/D program (46, 47). The experimental phases are sufficient for us to build the DNA and protein model with the aid of the Buccaneer program in the *CCP4* suite (Research Complex at Harwell [RCaH] STFC Rutherford Appleton Laboratory) and *Coot* (*https://www2.mrc-lmb.cam.ac.uk/personal/pemsley/coot/*). Structural refinement was performed with *PHENIX*. *Refine* program (47, 48, 49). ZBTB10 ZF1–2^R767Q^ was then solved with MOLREP in the *CCP4* suite employing the ZF1–2 structure as a search model. The crystallographic parameters are displayed in Table S2. All the structures in the figures were generated using PyMOL (DeLano Scientific LLC).

### ITC

ITC assays were performed at 20 °C by using a Microcal PEAQ-ITC instrument (Malvern). Double-stranded oligo deoxynucleotides are added to the sample pool at a concentration of approximately 30 μM, and proteins are added to the syringe at a concentration of approximately 300 μM. A typical ITC experiment consisted of 19 drops, with one injection of 1 μl followed by 18 injections of 2 μl of protein sample. The integrated heat data were analyzed using a one-site binding model by MicroCal PEAQ-ITC Analysis Software provided by the manufacturer.

## Data availability

The atomic coordinates and structure factors for the ZBTB10 ZF1–2/TTGGGG and ZBTB10 ZF1–2^R767Q^/TTAGGG structures have been deposited into the Protein Data Bank under accession codes 8GN3 and 8GN4, respectively.

## Conflict of interest

The authors declare that they have no conflicts of interest with the contents of this article.
